# Investigation of Mechanical Strains in Thermal Compensation Loop of Superconducting NbTi Cable during Bending and Cyclic Operation

**DOI:** 10.3390/ma14051097

**Published:** 2021-02-26

**Authors:** Artur Iluk

**Affiliations:** Faculty of Mechanical Engineering, Wrocław University of Science and Technology, Łukasiewicza 7/9, 50-371 Wrocław, Poland; artur.iluk@pwr.edu.pl

**Keywords:** superconducting cable, NbTi superconductor, thermal compensation, numerical simulation, bending strain

## Abstract

In the paper, the thermal compensation loops on a composite, superconducting NbTi cable were investigated. This type of cable is used in the superconducting, fast ramping magnets of the SIS100 synchrotron, part of the Facility for Antiproton and Ion Research (FAIR) under construction in Darmstadt, Germany. The influence of space restrictions and electromagnetic cross-talk on the design of the thermal compensation loop was discussed. Plastic deformation of cable components during bending was analyzed by numerical simulations and experiments. A three-dimensional numerical model of the cable was prepared with individual superconducting wires in contact with a central cooling pipe. The bending of a straight cable into a compensation loop shape was simulated, followed by cyclic operation of the cable during thermal cycles. The maximum strains in the superconducting strands and cooling tube were analyzed and discussed.

## 1. Introduction

**Figure 1:**
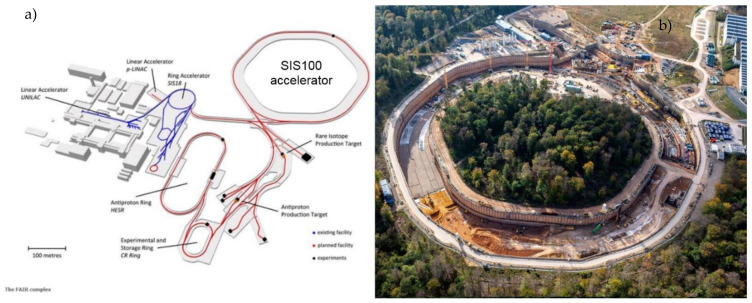
The layout of the FAIR facility (**a**) [5] and bird’s eye view of the SIS100 construction place (**b**).

The Facility for Antiproton and Ion Research (FAIR) is expected to be one of the most complex international accelerator facilities for the research of antiprotons and ions, currently under construction at the GSI Helmholtzzentrum fur Schwerionenforschung near Darmstadt, Germany. The central part of the facility is a SIS100 synchrotron, with a circumference of 1100 m, associated with a complex of the experimental setups (Figure 1).

The electric current is transferred to the magnets of the SIS100 synchrotron through a superconducting transfer line, simultaneously providing the cooling helium for the magnets. The transfer line design is unique, because, in a standard installation, the cooling helium and electrical power are transferred separately [1,2,3]. In SIS100, the electrical busbars and hydraulic process pipes coexist in a single, relatively small vacuum vessel. More details about the SIS100 design are provided in [4,5].

The superconducting busbar used for the transfer of the electrical current and simultaneously in the design of superconducting magnets is based on a hollow cable cooled with a forced two-phase helium flow [6,7,8]. The cable type was developed as an optimal solution preventing damage of the coils by fast-pulsing loads over the lifetime taking into account the complex fine structure of the cable and coil designs as well as its sensitive influence on the field quality, AC loss generation and quench protection [9]. The design of the cable is based on a similar cable used in the Nuclotron accelerator in Dubna, Russia [8,10].

Although elastic superconductors with higher critical temperature like MgB_2_ have been developed [11], the NbTi superconductors are still used due to good electric and mechanical properties [12]. The modern application of this type of superconductor, except for the FAIR project, was described in [13,14].

In the paper, the thermal compensation loop on a composite superconducting NbTi cable was investigated. Several technical aspects influencing the design of the loop were presented. The maximum strains introduced at the manufacturing stage into the superconducting strands and cooling tube were analyzed and discussed.

## 2. Design of the Busbar Compensation Loop

In the SIS100 transfer line, the four busbar pairs are arranged around the helium process pipes as far from each other as possible in order to minimize electromagnetic cross-talk between busbars, affecting the quality of the magnet control. Another aspect influencing the design of the busbar layout was the eddy current generation in the busbar system [15,16].

**Figure 2:**
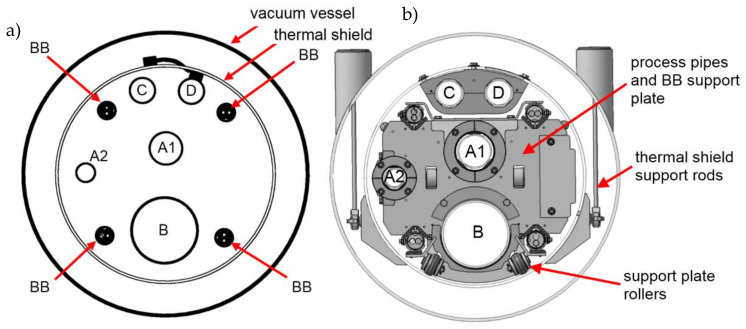
General cross-sectional layout of SIS100 DN450 bypass line with coexisting helium process pipes and superconducting busbars: without supports (**a**), with support system (**b**). A1, A2, B, C, D—helium process pipes, BB—superconducting busbars.

The length of the longest single section of the transfer line is 12.9 m. Due to the thermal contraction of the busbar at temperature 4 K, the thermal compensation system should be able to compensate 40 mm of the thermal contraction of the busbars from room temperature to 4 K. The general cross-section of the transfer line is presented in Figure 2.

The direct consequence of combining the helium process pipes and superconducting busbars into a common straight vacuum vessel was the necessity of providing thermal compensation on the busbars, with a similar function as the axial compensators on the process pipes, within a very limited space. It makes several challenges in the design:

- selection of the optimal shape for compensation loops in limited space,
- minimizing of the electromagnetic cross-talk between busbar pairs,
- possible excessive plastic strain in superconducting strands during busbar bending process at the manufacturing stage,
- possible excessive strain in superconducting strands during thermal cycling between 3000 and 4 K.

The first two challenges are connected directly with the shape (design) of the compensation loop. In the traditional cryogenic transfer line, the thermal contraction of the process pipes, approximately 3 mm per meter, is compensated by corrugated bellows [1]. The thermal contraction of the superconducting cable, similar to the contraction of the process pipes, has to be compensated by the cable geometry, for example, by compensating loops.

The distance between axes of the busbar pairs is 195 mm and the compensation loops have to fit in space between pairs. The minimal recommended bending radius was 25 mm, however, this was stated for stationary work. Because of nonstationary bending during thermal cycling, the bending radius should be increased. Space was restricted not only in the transversal direction; the length of the loop in the axial direction had to be minimized as well.

**Figure 3:**
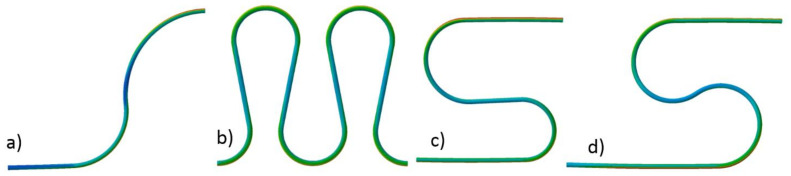
Investigated shapes of the thermal compensation loops. Due to symmetrical movement, only left half of the real loop is presented. (**a**) single vertical loop, (**b**) four vertical loops, (**c**) single omega-shaped loop with two different diameters of bending, (**d**) single omega-shapeed loop with constant diameter of bendind.

The structure of the cable makes it highly flexible and suitable for bending with a relatively small radius. The topic of the formability and maximum allowed strains was not discussed in the existing literature, except for several papers dealing mainly with the magnet design and mechanical behavior of the cable inside the magnet structure [17,18]. In some applications in the magnet design, the minimum bending radius of the cable was claimed to be as low as 17.5 mm [17]. The various shapes of the compensation loops were checked by numerical simulation (Figure 3) for the ability to compensate 40 mm of the thermal contraction in available space and with minimum strain. The shapes were modeled as simply pipe with the geometry of the cooling tube with the use of shell finite elements.

For all shapes, with applied displacement, the maximum strain was checked. Finally, the shape presented in Figure 3d was selected due to the lowest strain (highest flexibility) in a limited space. The shape presented in Figure 3b exhibited similar flexibility but was rejected because it requires a more complicated mechanical stabilization system.

**Figure 4:**
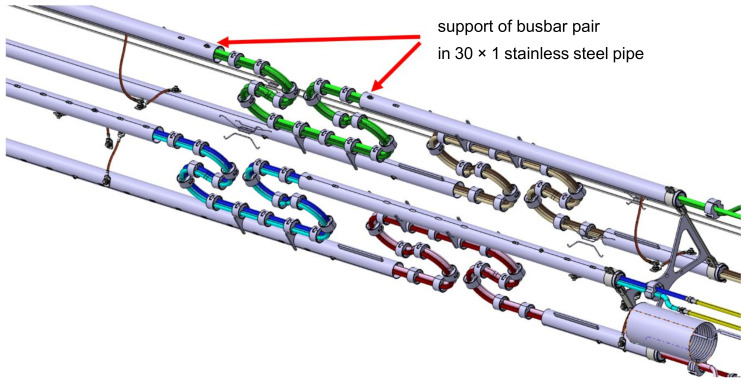
View of compensation loop area of the SIS100 bypass line.

The compensation loops are present on all four busbar pairs. Due to limited space, all loops have to be designed in the vertical plane and located close to each other to fit in the same space where the process pipes are located. It means that in the loop area, the busbars will be close to each other, increasing the electromagnetic cross-talk. The view of the 3D model of the compensation loop region is presented in Figure 4.

**Figure 5:**
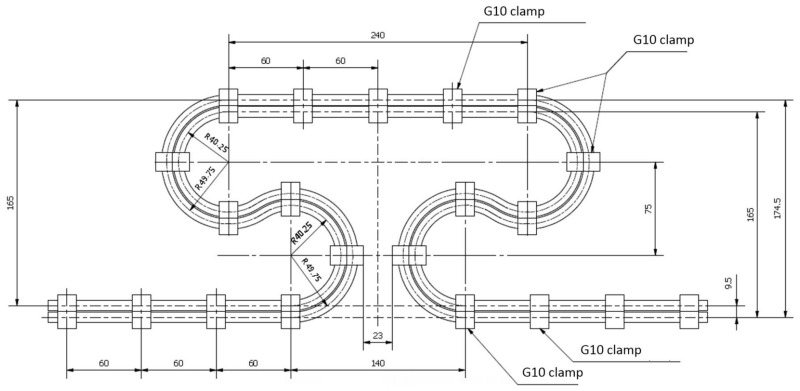
The vertically oriented, non-twisted busbar orientation of the loop routed in a vertical plane (not used in design)—results in very high stiffness and lack of compensation ability, dimensions in (mm).

The orientation of the busbar pair in the loop area is essential from a mechanical and electromagnetic point of view. The busbars in every pair are clamped every 60 mm to carry out the Lorentz force 3.81 kN/m in value. In order to keep the high flexibility of the clamped busbar pair, it must be oriented horizontally in the area of the loop. A vertically oriented pair, shown in Figure 5, has a much higher moment of inertia of the cross-section and is much stiffer, making the compensation loop useless.

**Figure 6:**
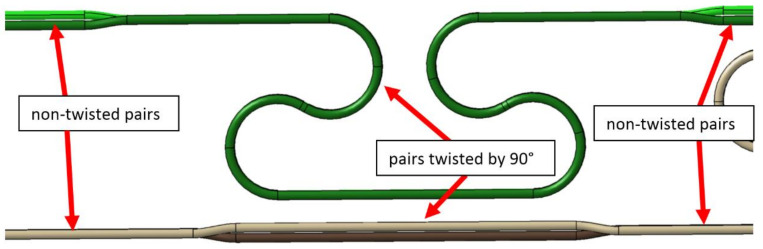
Twisted busbar pairs in the area of the compensation loop. The upper busbar pair is twisted to minimize mechanical stiffness, the lower busbar pair is twisted to minimize the electromagnetic cross-talk.

In the standard cross-section area (Figure 2), two busbar pairs are oriented vertically, so have to be rotated by 90° to the horizontal orientation in the whole loop area. Unfortunately, the upper branch of the loop is routed there very close to the other straight busbar pair, with horizontal orientation too. Closely located, parallel busbar pair with the same orientation is the worst case from the electromagnetic cross-talk point of view. For this reason, the upper straight busbar pair was also rotated by 90° to make the orientation of both pairs perpendicular and minimize cross-talk (Figure 6).

For the presented design of the busbar compensation loop, the mechanical strain in the superconducting wires (strands) was investigated. The cable and its wires have to be plastically deformed during the loop formation process, and later in normal operation, the loop is periodically deformed by the thermal contraction.

The strain is an important factor influencing the ability of superconductors to transfer an electrical current [19,20,21]. The critical current I_c_—current at which the thermal runaway of a superconducting strand (so-called quench) occurs—depends mainly on the magnetic field the superconductor is working in, the temperature and mechanical stress applied to the superconductor [22,23]. While for the superconducting high field magnets used in accelerators the more important restriction is the magnetic field intensity, for the busbar in the transfer line more significant is the mechanical strain in the thermal compensation loops.

The strain state of superconductors has an influence on the ability of current transfer. The critical current I_c_ depends mainly on the magnetic field and the rise of temperature or strain causes the so-called quench when the superconductor rapidly falls into the normal conducting state [24].

The critical current limitation was investigated by many researchers, but mainly in the context of superconducting magnet windings and high magnetic field application. In [25], the significant influence of the fast magnetic pulses on the critical current was described. The influence of microstructure and grain boundaries on the critical current was described in [26,27]. The negative correlation between the grain boundaries misalignment angle and the critical current was stated.

Another important factor influencing the critical current is mechanical strain. The fatigue and bending strain influence on the critical current in thin-layer Ti/Nb/Cu and Nb-only superconductors was investigated in [28]. The reversible response of I_c_ with uniaxial strain in differently processed GdBCO coated conductor tapes with different substrates under self-field and magnetic field conditions at 77 K was investigated in [29]. It was found that the critical current drops significantly at the strain of 0.6%. The effect of axial strain on the critical current in a high-temperature superconductor for electric power application was studied in [30]. It was presented that the strain 0.33% significantly limits the critical current.

**Figure 7:**
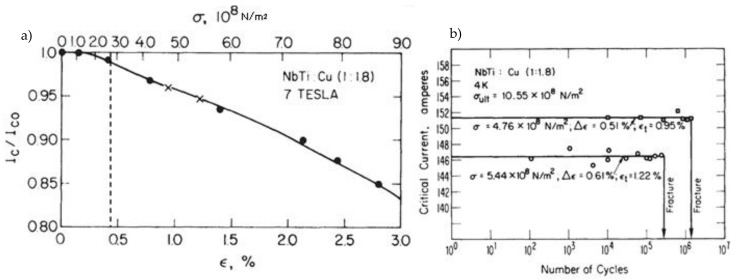
Degradation of critical current in NbTi strand as a function of strain (**a**) and number of cycles to fracture for different cyclic strain levels (**b**) [32], the strain level obtained in presented research was marked with dash line.

The multifilament NbTi strands were investigated in [31,32]. The influence of strain on the critical current for magnetic field 7 T and fatigue resistance of the strand are presented in Figure 7. In the figure, the level of plastic strain from the current work was marked.

Due to the complex geometry of the superconducting strands wrapped around the busbar in the area of the compensation loop, in order to check the actual strains in the superconducting strands, a numerical model of the complete busbar was developed and subjected to a numerical simulation of the bending process. In the paper, the maximum strains induced by the manufacturing process and operational movements in cable components were investigated.

In the existing literature, there is no investigation of strain in individual strands of Nuclotron-type cable during bending. In [33], the influence of bending of individual wire on the conductivity of a single NbTi wire was investigated. It was found that total strain up to 0.025 is not degrading the conductivity significantly.

In the paper, the strains developed during bending and operation in the superconducting wires wound around the central tube were investigated. Additionally, the design of the thermal compensation loop for the busbar system in the SIS100 bypass line was presented.

## 3. Preliminary Tests and Simulations

**Figure 8:**
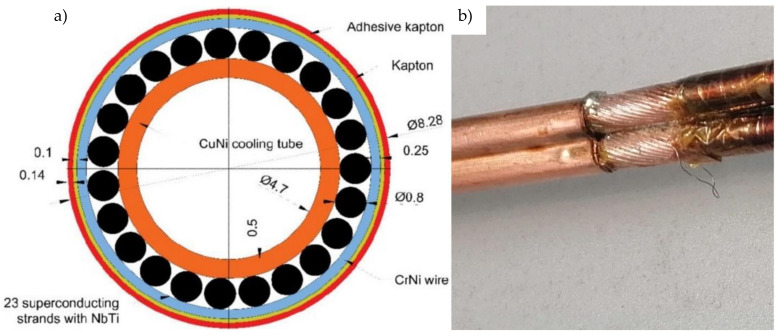
(**a**) Cross-section of the NbTi superconducting cable for the busbars of SIS100 [16] (**b**) and the real superconductor uninsulated and soldered.

The internal structure of the superconducting NbTi cable is presented in Figure 8. The central tube with a diameter of 5.7 mm made of CuNi alloy is actively cooled by liquid helium flowing through its cross-section. Around the central tube are wrapped 23 superconducting NbTi strands with diameters of 0.8 mm with pitch equal to 47 mm. Each strand is a composite of superconducting NbTi filaments in the copper matrix. In order for the strands to stay in contact with the central tube during bending, the strands are wrapped with 0.5 mm wire. The cable is electrically insulated by two layers of Kapton foil. The main parameters of the superconducting cable used in the SIS100 bypass line are presented in Table 1.

## 4. Mechanical Properties of Cable Components

The mechanical properties of the busbar components were measured experimentally with the tensile test machine Zwick&Roell (Kennesaw, GA, USA). The strain–stress characteristics of the CuNi central tube and superconducting wire were obtained at room temperature. The obtained curves were used later for simulation purposes.

### 4.1. Superconductor

The chemical composition of the superconducting NbTi checked with the use of a spectrometer is shown in Table 2. For the tensile tests, the wire was extracted from the cable, straightened and 150 mm long samples were cut. The tensile tests were carried out according to the ISO 6892-1 with a controlled strain rate of 10^-4^ 1/s. The strain was measured with the use of a mechanical extensometer, with a base distance of 50 mm.

**Figure 9:**
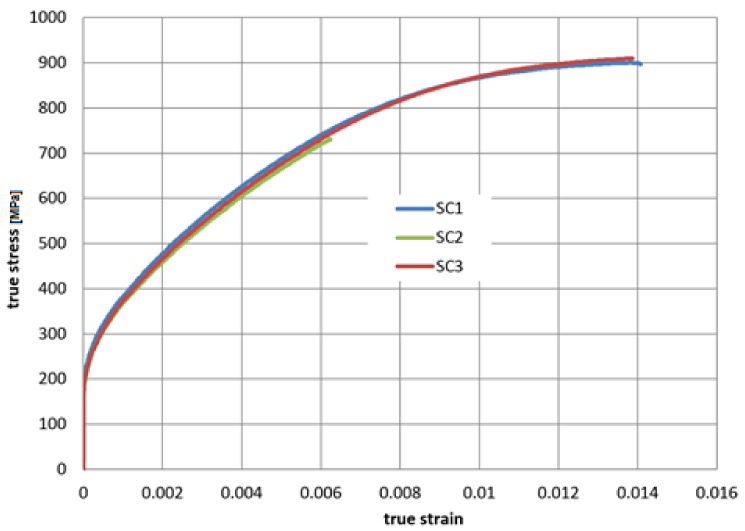
The true stress–true plastic strain characteristics of three superconducting wire samples in tension.

The mechanical properties of the wire from the tensile test are presented in Table 3. The nominal stress-strain curve was converted to true stress and true strain. After removing the elastic part of strain calculated from the Young modulus, the characteristics suitable for the numerical plasticity model of the material are presented in Figure 9. The shapes of all curves are similar, and sample 2 broke earlier at the clamp, due to difficulties with clamping of the wire without stress concentration at the clamp.

### 4.2. Central Cooling Tube

The chemical composition of the central cooling tube checked with the use of a spectrometer is shown in Table 4. For the tensile tests, the tube was extracted from a straightened cable. On the 150 mm long sample, the tube was filled out in the clamping areas and clamped on the test machine. The tensile test was carried out according to the ISO 6892-1 with a controlled strain rate of 10^-4^ 1/s. The strain was measured with the use of a mechanical extensometer, with a base distance of 50 mm.

**Figure 10:**
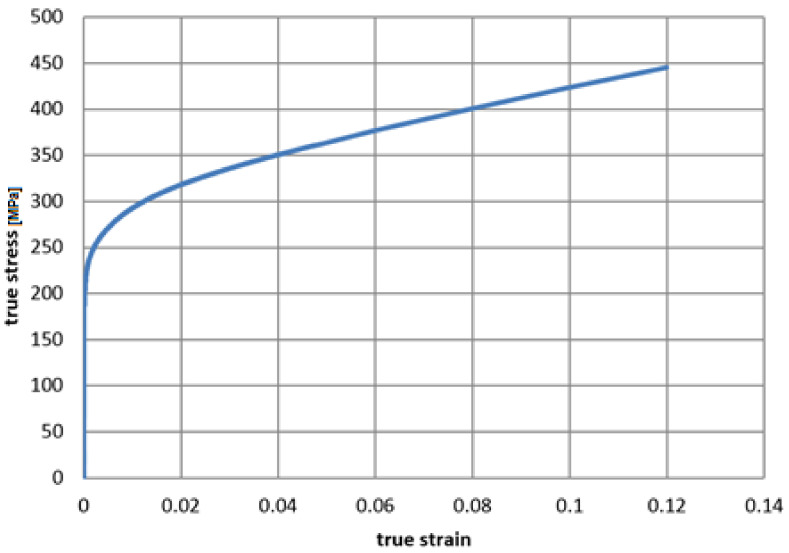
The true stress–true plastic strain characteristics of cooling tube sample in tension.

The mechanical properties of the tube from the tensile test are presented in Table 5. The nominal stress-strain curve was converted to true stress and true strain. After removing the elastic part strain calculated from the Young modulus, the characteristics suitable for the numerical plasticity model of the material are presented in Figure 10.

In order to check the response of the superconducting cable to bending load, dominating in the case of the thermal compensation loops, the simple loop was investigated in the cyclic load in symmetrical cycles with increasing amplitudes up to ± 30 mm with the constant speed of 1 mm/s. The displacement was controlled, and the force was measured.

**Figure 11:**
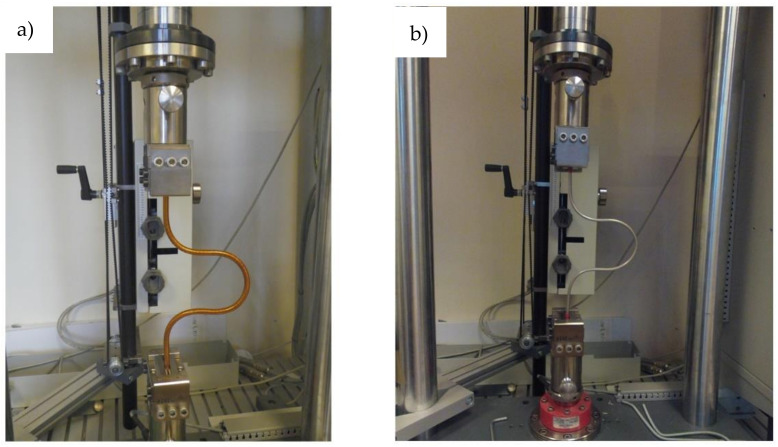
(**a**) The measurement of the mechanical properties of the complete busbar and (**b**) the cooling tube with stripped off superconducting wires.

The geometry of the sample contained four arcs with radiuses equal to 45 mm, the same as in the final geometry of the loop. The loop made of complete cable and the loop of the same geometry but containing only the cooling tube without superconductors were investigated. The view of both loops on the tensile machine is presented in Figure 11.

The reason for testing the whole cable was to check the hysteresis due to friction of the full system containing superconducting wires, cooling tube, external CrNi wire and Kapton insulation. The bare cooling tube was tested to detect the point at which the hysteresis starts to increase due to leaving the elastic range of material characteristics. It allows us to separate the material hysteresis of the cooling tube from the whole cable.

**Figure 12:**
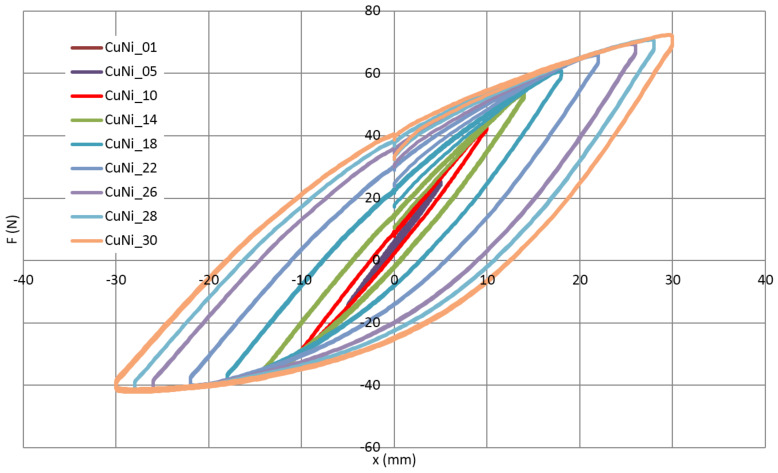
The force (F)-displacement (x) characteristic of the cooling tube with increasing displacement amplitude from 1 to 30 mm.

**Figure 13:**
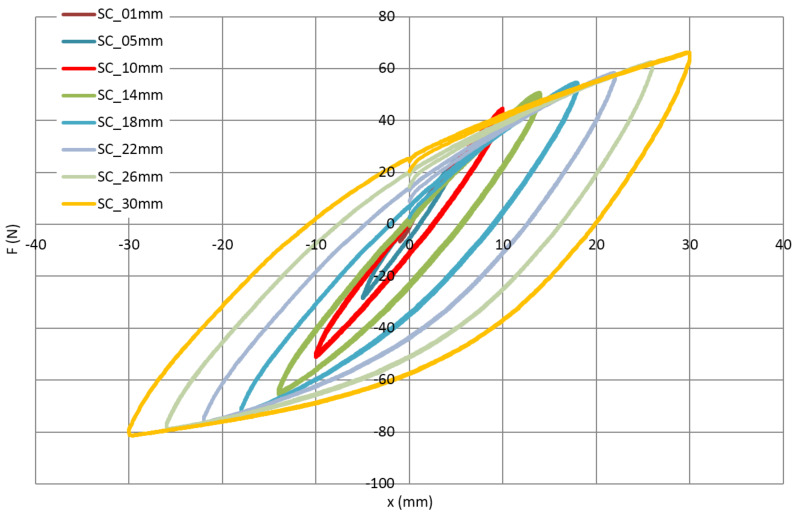
The force (F)-displacement (x) characteristic of the complete superconductor with increasing displacement amplitude from 1 mm to 30 mm.

A mechanical hysteresis of the cooling tube and the whole cable are presented in Figure 12 and Figure 13, respectively. The hysteresis visible in Figure 13 (complete cable) has two sources. The first is the friction between the central tube, superconducting wires, NiCr external winding and insulation made of two Kapton layers. The second is increasing plastic deformation of the metallic components of the cable. In the case of the bare cooling tube (Figure 12), only the second effect is present. The difference between both figures can be interpreted as an influence of friction on the energy dissipation during cycles.

The results of the bending test show that for amplitudes up to approximately 10 mm, the mechanical hysteresis is relatively low. It corresponds to the work of loop in the elastic range, the main source of energy dissipation is friction. In the case of the cooling tube test, it is almost the straight line. Above the amplitude of 10 mm, the dissipation starts to increase due to plastic deformation.

It should be highlighted that the compensation ability of the loop is proportional to the number of bends. The tested simple loop has the same bending radius as the loop designed for SIS100 and a twice-smaller number of bends. This means that amplitude +/−10 mm in the tested loop corresponds to amplitude +/−20 mm for the SIS100 compensation loop, which is the maximum amplitude in the real condition. This means that the selected shape of the compensation loop is able to fully utilize its ability to compensate for the thermal contraction of the busbars.

## 5. Numerical Simulation of Manufacturing and Operation

As stated before, the mechanical strain in the superconducting has a significant influence on the critical current. In order to check the strain state in the thermal compensation loop, a detailed numerical model of the superconducting cable was prepared. In the existing papers, there are a few publications about FEM modeling of the NbTi superconducting cables. The trial to make a detailed 3D model of SIS100 NbTi cable was described in [18], but without any results. The single wire of NbTi superconductor with internal filament structure was investigated in [34] by the numerical simulation of the electromagnetic fields to estimate the critical current, but without considering the entire cable structure and neglecting the mechanical strain. A similar investigation, but connected with the plastic strain during bending and subsequent thermal load in the cooling tubes of the Wendelstein 7-X nuclear fusion experiment was described in [35].

A very common simplification in numerical modeling is the preparation of the geometrical and discrete model in the shape defined at the manufacturing stage. All operations, even including a significant plastic deformation made during the manufacturing process are just neglected and the material is treated as a raw one. It is correct only if the material is annealed after plastic deformation. Otherwise, especially for metallic materials, the history of plastic deformation can have a significant influence on the stress state due to the strain hardening phenomenon. This effect is, for example, considered in the advanced crash test simulation of cars. The components of the car structure are modelled with included residual strains from the stamping stage [36].

In the case of the compensation loop on the superconducting cable, the strain in the superconducting wire has three components:

- strain induced by the cable production—relatively low because of the high pitch of the helix,
- strain induced by the loop bending process—most significant component due to relatively small bending radius,
- cyclic strain during thermal cycling of the loop—preferably in the elastic range only.

The first component, the strain of cable manufacturing, is very difficult to include in the model. It requires a simulation of the full wire production process. The second component, the plastic strain introduced during bending of the loop, is potentially the most significant. It was introduced in the simulation model by simulation of the loop bending process.

The numerical model of the busbar was prepared in the implicit finite element code ABAQUS/Standard. To obtain the final strain in all wires of the compensation loop, a full process of bending starting from the straight busbar was simulated. This approach ignores the plastic strain introduced during the manufacturing of the cable, but because of the relatively long pitch of the spiral of wire equal to 47 mm, the strain introduced at this stage is small in comparison to the loop bending strain and can be neglected. The small plastic strain introduced into the cable at the manufacturing stage was confirmed by the relatively low plastic deformation of the superconducting wires disassembled from the straight cable.

**Figure 14:**
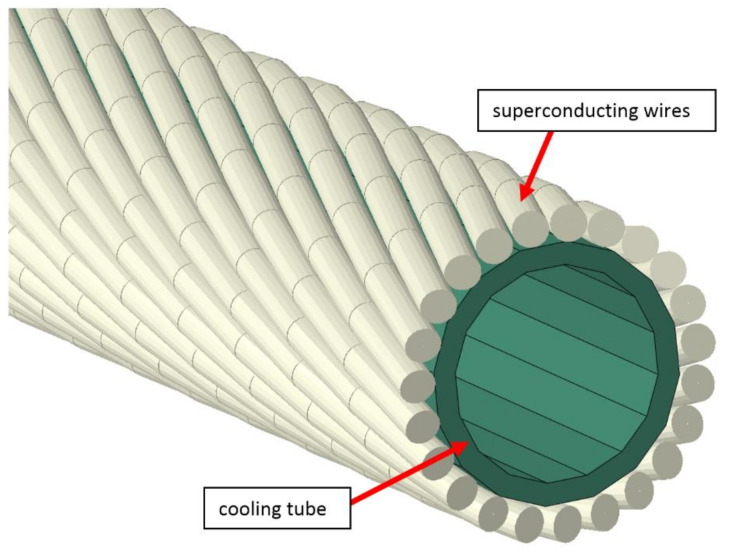
The discrete model of the busbar.

The numerical model of the central tube was prepared with 7488 I-order continuum shell elements [37] of average size 1 mm. Superconducting wires wrapped around the central tube were modelled with the use of 5474 Timoshenko shear-flexible beam elements of an average size of 3 mm. The friction between copper and copper was reported in [38] in the range 0.25–0.6. Contacts between wires and central tube and between the wires were defined with the friction coefficient equal to 0.3, however, the simulations were conducted as well for the friction coefficients up to 0.6 without significant changes in the results. The presence of an external 0.5 mm wire preventing the separation between wires and the central tube was simulated by switching off the ability of the contact wire-tube to separate, only lateral relative movement was allowed. The view of the busbar discrete model is presented in Figure 14.

The isotropic elastoplastic material models with isotropic hardening were used for the superconductor and cooling tube. The Young modulus and true stress—true plastic strain characteristics were adopted from the experimental test results of the superconducting wires (Table 3, Figure 9) and the cooling tube (Table 5, Figure 10).

During the whole simulation, one side of the busbar was fixed (all six degrees of freedom—DOF) at the point wherein the real compensation loop is supported in the support 30 × 1 pipe (shown in Figure 4).

**Figure 15:**
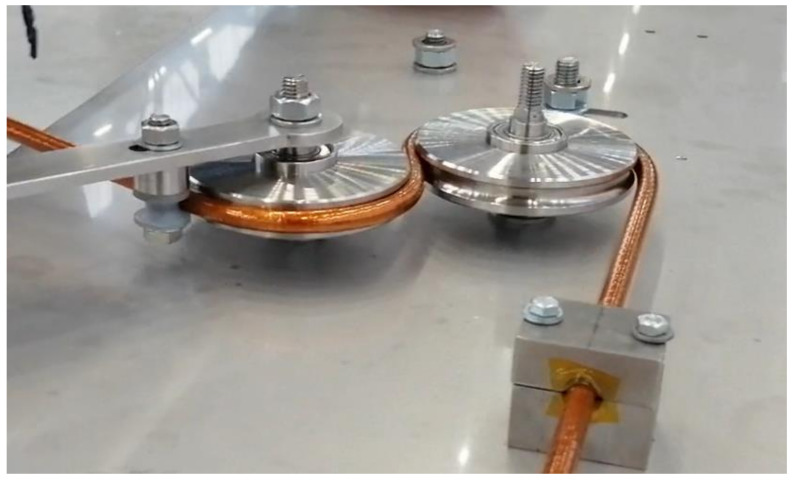
The compensation loop manufacturing, bending of busbar on the rigid rollers.

To resemble a real bending process, a set of bending rollers was introduced to the model. The rollers are modelled as a perfectly rigid body. The bending process kinematics definition was not a trivial task, because to achieve the required final shape of the compensation loop, the elastic springback must be taken into account. The figure of the real manufacturing stand during the loop bending is presented in Figure 15. Due to the complexity of the busbar design, the required angles of subsequent bending actions were found iteratively.

**Figure 16:**
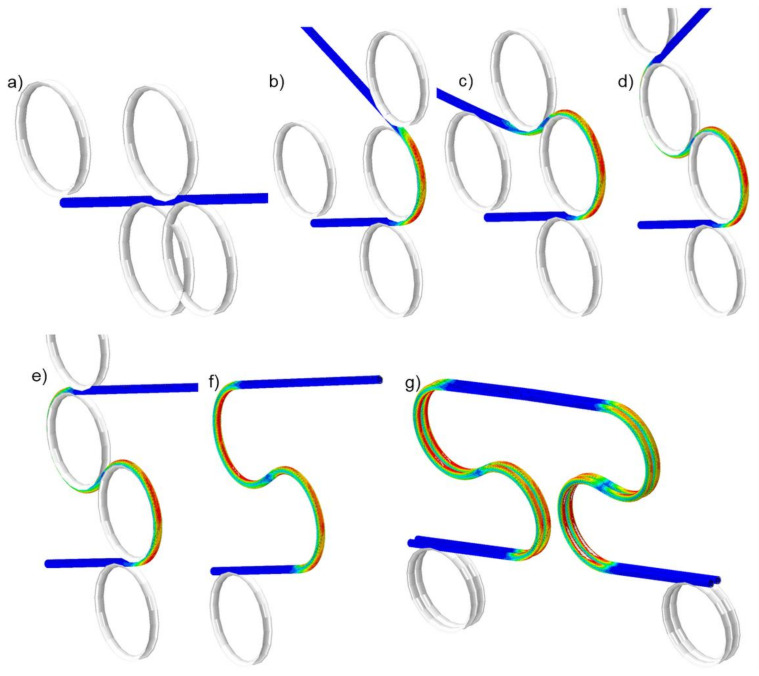
The stages of the busbar compensation loop bending process: (**a**)—straight cable, (**b**)—bending of the first loop, (**c**,**d**)—bending of the second loop, (**e**)—final stage of bending before springback, (**f**)—final shape after springback (removed rollers), (**g**)—assembled busbar pair, ready for cyclic work.

At the final stage, the bending rollers were removed from the simulation. The shape of the compensation loop after the final springback fits required design geometry. The subsequent stages of the bending process are shown in Figure 16 and provided as an animation in the Supplementary Materials.

The simulation of bending, due to strong geometrical nonlinearity and presence of many contacts was very time-consuming. For this reason, the symmetry of the compensation loop and deformation shape was utilized and only half of the full loop was prepared. In the next stage, the deformed model with preserved strain state was mirrored and subjected to cyclic axial displacement, resembling thermal cycles of cool down to 4 K and warm up to room temperature. It was realized by kinematic axial movement of the supported endpoint of the loop (Figure 4) by +/−20 mm, with all other DOF fixed.

The whole simulation was realized as a nonlinear dynamic simulation to avoid problems with the convergence of the solution. Each of the bending actions was divided into at least 100 increments and each of the thermal contraction and expansion cycle was divided into at least 20 increments. Due to the automatic incrementation algorithm and complex contact system between wires and central tube, especially during bending, the total number of increments in simulation exceeded 1000.

## 6. Results of Simulation and Discussion

**Figure 17:**
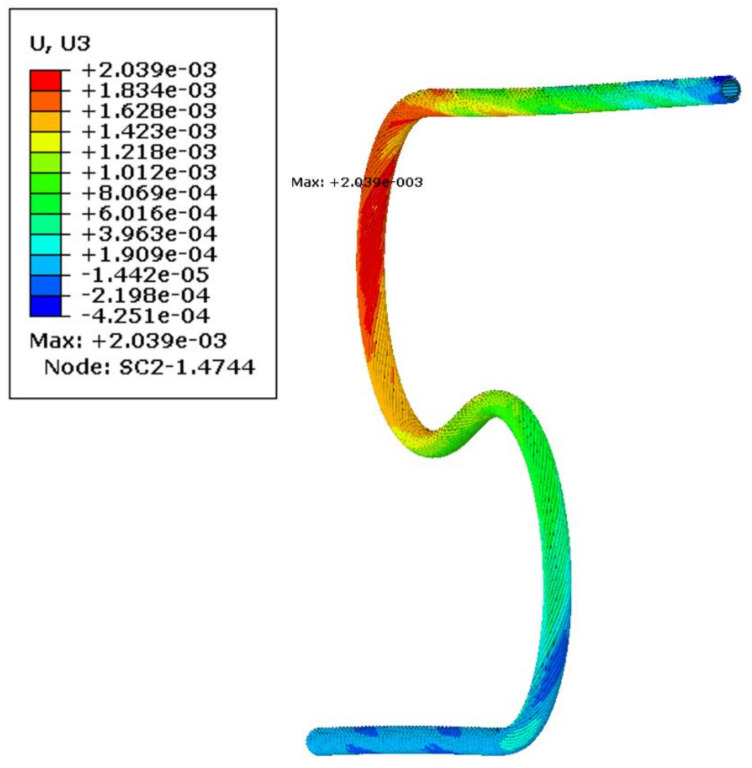
Out-of-plane (lateral) displacement after bending and springback (m).

It was found during simulation, that due to the spiral shape of wrapped superconducting wires, the bent busbar had a significant tendency to leave a bending plane (Figure 17). This interesting effect of the out-of-plane deformation was observed later when a real compensation loop was manufactured. The reason for deformation is the spiral shape of superconducting strands.

**Figure 18:**
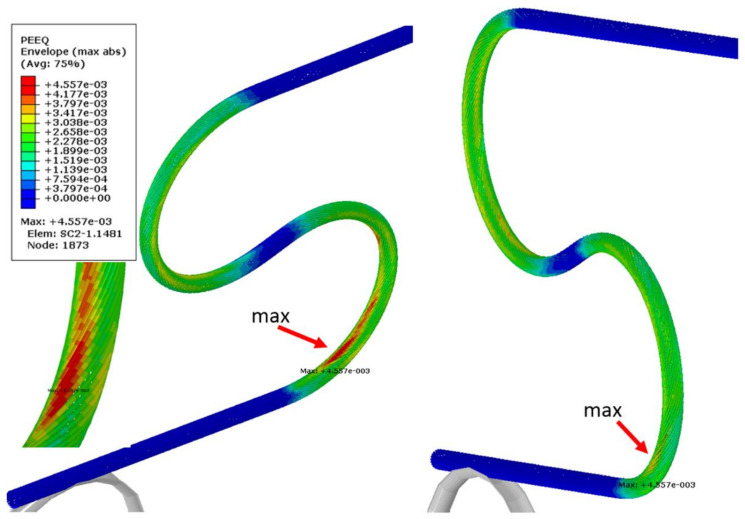
Maximum equivalent plastic strain in superconducting strands after bending and springback.

**Figure 19:**
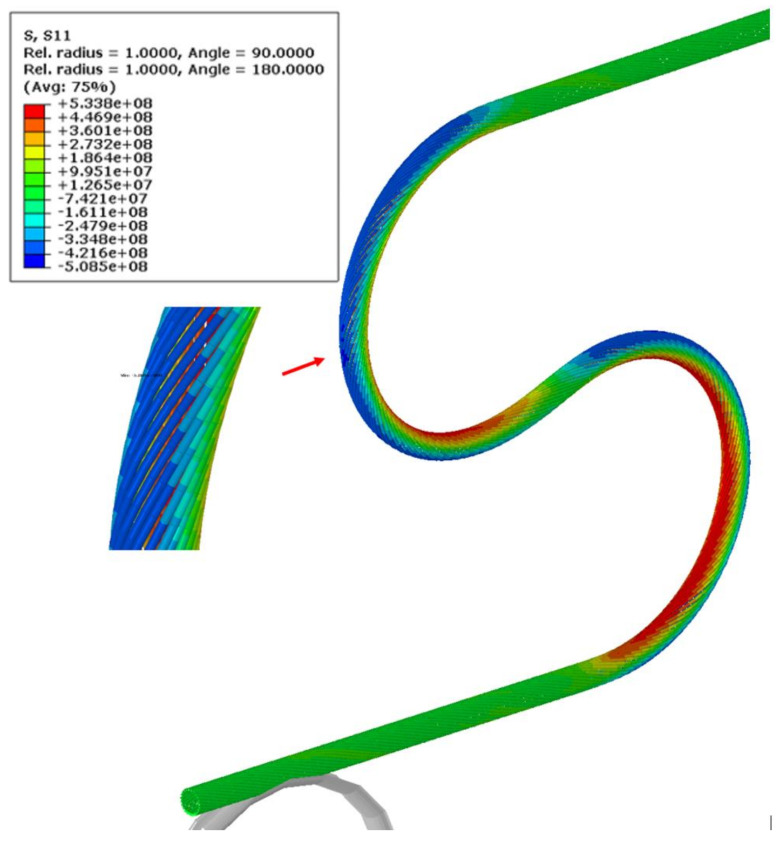
Axial stresses in the superconducting wires at the end of bending process (Pa).

The most deformed areas of the superconducting wires are presented in Figure 18. It is located at the onset of the first bend. In Figure 19, the region of maximum axial stress is shown.

**Figure 20:**
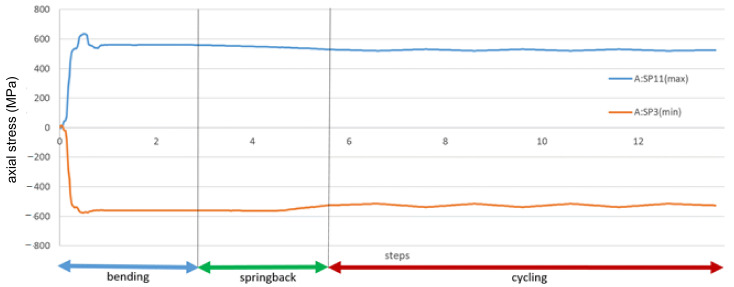
History of stress of the most deformed areas of the superconducting wires from Figure 19. The stages “bending” and “springback” correspond to manufacturing phases from Figure 16.

In Figure 20, the history of axial stress and plastic strain during bending, springback and cycling for the maximum strain location are presented. The steps on the horizontal axis correspond to subsequent stages of the numerical simulation, not relevant for the results. For clarity, the entire simulation was divided into stages: bending, springback and cycling. The axial stresses are taken from two points at the same location on the wire but on two opposite points on the wire circumference—positive in tension.

The difference between the stresses corresponds to the bending. The tension and compression stresses are very similar in value and opposite in sign, which means that means axial stress is insignificant and the bending stress strongly dominates on the superconducting wires.

The most significant plastic strain is introduced during the bending stage, when the bending roller passes by the given location on the wire. The maximum plastic strain value detected is equal to 0.45%. As can be seen in Figure 7, this level of plastic strain decreases the critical current only by 1%.

The stress reaches the maximum value at the same time. After the passage of the roller, the plastic strain remains unchanged until the end of the simulation, while the stress is decreasing slightly during the rest of the bending process. The stress during cycling is lower than during bending. The amplitude of stress during cycling is very small in comparison to the bending stress.

**Figure 21:**
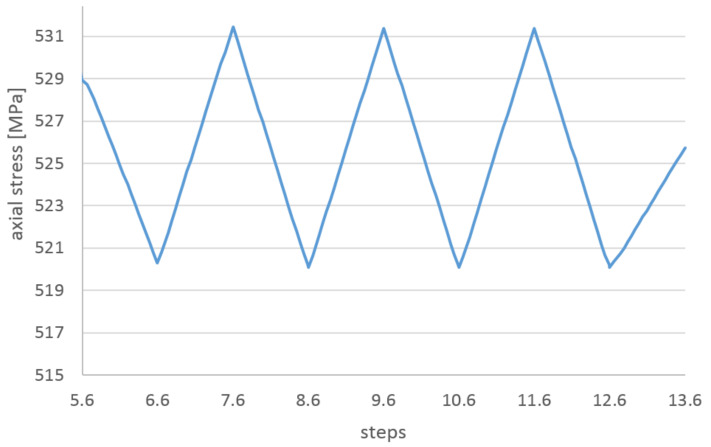
The axial stresses on the tension side of wire during cycling stage.

In Figure 21, the axial stresses on the tension side of the wire during the cycling stage are presented. The range of the cycling stress in the wire is only 10 MPa, very small in comparison to the yield stress. A low level of the stress range is confirmed by constant values of the plastic strain during cycling.

**Figure 22:**
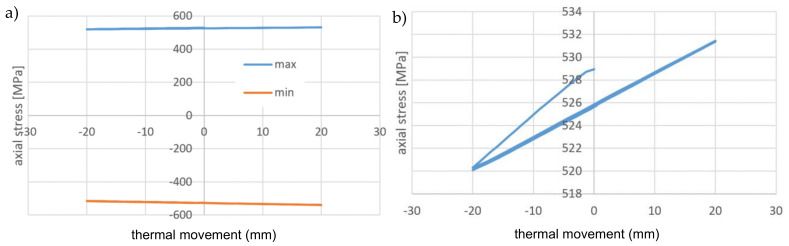
(**a**) The axial stresses during cycling stage as a function of the cycling displacement: on the tension and compression sides of wire, (**b**) on the tension side of wire.

In Figure 22, the axial stresses as a function of the cycling displacement are presented. There is no visible hysteresis due to the small amplitude of stress. The model is not able to resemble the friction between the superconducting wire external NiCr wire and the external Kapton insulation (not present in the model) but the friction wire-cooling tube is present. The lack of hysteresis means that the local movement wire-tube is small and does not dissipate the energy.

**Figure 23:**
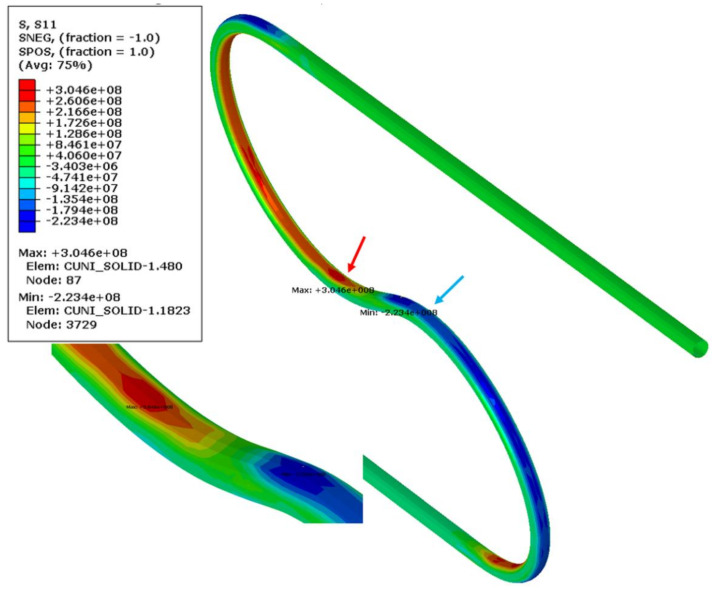
Minimum (blue arrow) and maximum (red arrow) axial stress in CuNi tube after bending and springback (MPa).

**Figure 24:**
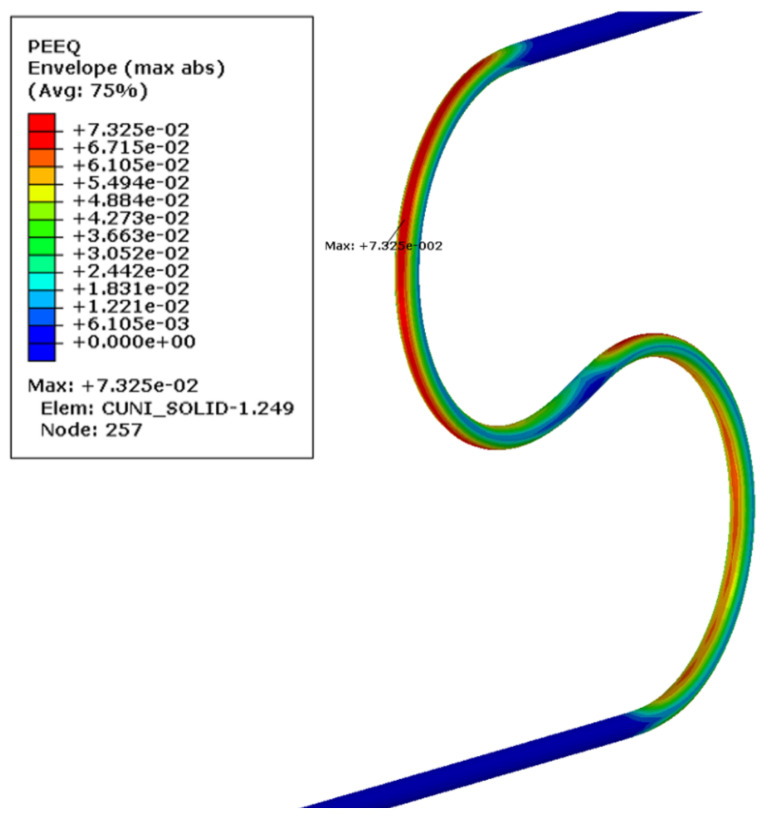
Maximum plastic strain in CuNi tube after bending and springback.

The axial stress and the plastic strain in the cooling tube were presented in Figure 23 and Figure 24, respectively. The plastic strain in the tube is one order higher than in the superconducting wire reached a value of 7.3%.

**Figure 25:**
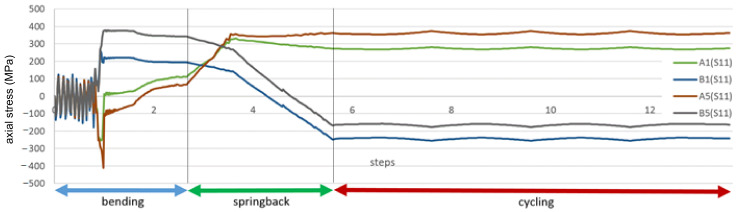
History of stress of the most deformed areas of the cooling tube from Figure 24. The stages “bending” and “springback” correspond to manufacturing phases from Figure 16.

The axial stress for the most stressed area of the cooling tube is presented in Figure 25. It behaves differently than the superconducting wire. While the stresses in wires after a rapid increase slightly decreases, the axial stress in the cooling tube changes the direction. The reason is the springback force applied to the central tube by plastically deformed wires. It means that not only the wires are more elastic (lower plastic strain) but also the bending stiffness of the superconductor wires is significantly higher than the bending stiffness of the central cooling tube.

**Figure 26:**
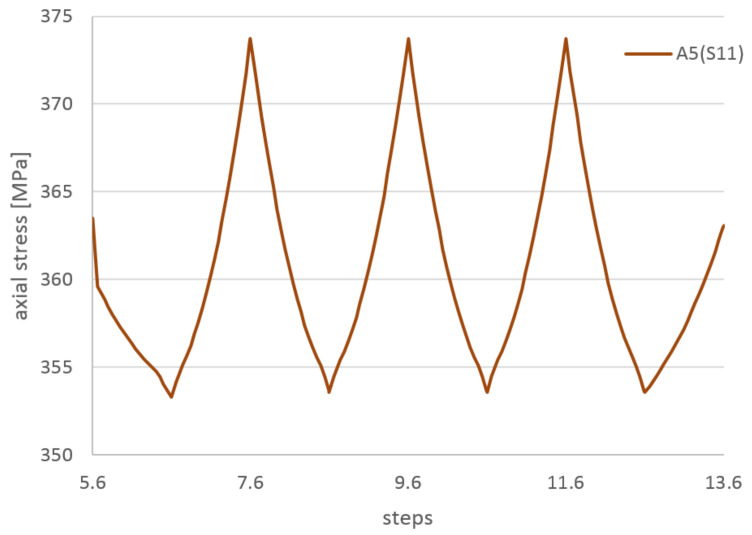
The axial stresses on the tension side of wire during cycling stage.

In the cycling stage, the axial stress range in the cooling tube is also small in comparison to the stress level. As shown in Figure 26, the stress range is equal to 20 MPa, which is twofold greater than the stress range in the wires.

**Figure 27:**
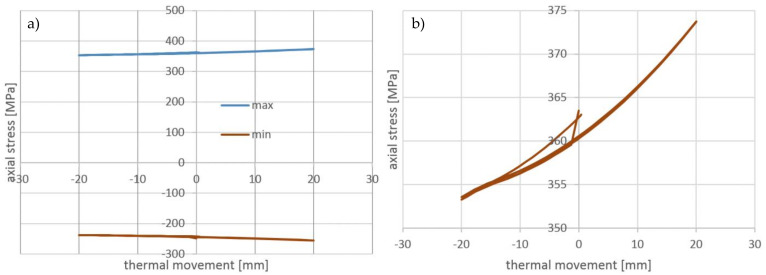
(**a**) The axial stresses during cycling stage as a function of the cycling displacement: on the tension and compression sides of wire, (**b**) on the tension side of wire.

The stress-displacement dependency for the cycling stage of the cooling tube presented in Figure 27 exhibits nonlinear behavior, however, there is no visible hysteresis. It confirms a lack of significant dissipation of energy due to friction.

The material models of all components were defined at room temperature because the main part of simulation, the compensation loop forming, takes place at this temperature. Subsequent cycles were not simulated by the cooling of the model, but by kinematically forced displacement of one end of the loop. The thermal contraction of the loop itself is negligible from the strain point of view, because the movement of the loop is free except for axial displacement.

The second part of the simulation, cooling down and thermal cycling, takes place in a real device at a temperature of 4 K. At this temperature, all components of the loop have slightly increased Young modulus by several percent [39]. However, the kinematic nature of the applied load also means that the stiffness has no influence on the strain and deformation of the entire loop. It will increase only the reaction force that was not extracted from the simulation. At 4 K, the yield stress of metallic material can be much higher than at room temperature [40]. As temperature-dependent material data were not available for all model components, a stress-strain curve at room temperature was used in the simulation. It was shown, however, that yield stresses were not exceeded during the thermal cycling, so they should be not exceeded for higher yield limits at 4 K.

The material data at room temperature used in the simulation are limitations of the presented simulation, but this simplification does not affect the most important results, the strains introduced by the manufacturing process and the strains during the thermal cycling. Including the temperature-dependent mechanical properties of all components and changing the busbar temperature by appropriate boundary conditions in the model could increase the precision of the stress prediction. However, with the yield stresses at a temperature of 4 K, significantly higher than at room temperature [40], the obtained results are conservative.

The maximum plastic strain detected in the superconducting wire was equal to 0.45%. After the manufacturing stage, it remains constant. The range of the stress in the superconductor during the thermal cycling is equal to 10 MPa. The average axial stress in the superconducting wire is insignificant in comparison to the bending stress.

For the central cooling CuNi tube, the maximum strain during the loop manufacturing stage reached 7.3%. The range of the stress in the cooling tube during the thermal cycling is greater than in the superconductor, and equal to 20 MPa. The bending stresses in the cooling tube change sign during the springback stage due to springback reaction forces of the superconducting wires. This means that the set of wires is more elastic (lower plastic strain) but also the bending stiffness of the superconducting wires is significantly higher than the bending stiffness of the central cooling tube.

## 7. Conclusions

In the paper, the design of thermal compensation loop for the superconducting busbar system of the bypass line, the part of SIS100 accelerator under construction in Darmstadt, Germany. The space restrictions and electromagnetic cross-talk influence on the actual geometry of the compensation system were discussed.

The preliminary experimental tests made on a simple loop confirmed that the designed compensation system should work without significant dissipation of energy within the elastic range.

For the designed compensation system, a numerical simulation was carried out in order to check the strain field in the components of the superconducting cable. For the final geometry of the loop, the strains introduced on the compensation loop manufacturing stage were taken into account by simulation of the bending process of the loop.

**Figure 28:**
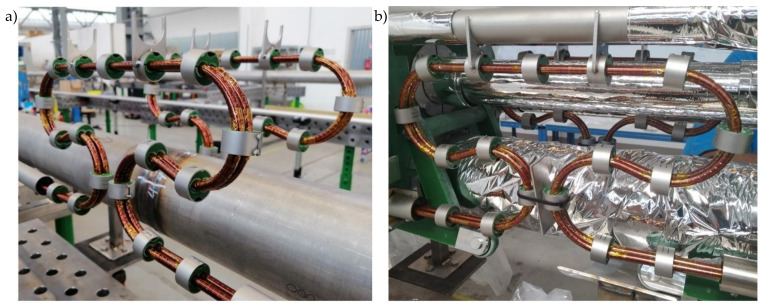
(**a**) The thermal compensation loop in the linear module of Cryogenic Bypass Line. The loops on the lower busbar pairs with the clamping system. (**b**) The compensation loops with the process pipes and thermal insulation.

The thermal compensation loop investigated in the paper was used in the modules of the Cryogenic Bypass Line (BPL) of the SIS100 accelerator. In Figure 28, the real view of compensation loops in the linear BPL module is presented. On the busbar pairs, the clamping system counteracting the Lorentz forces and process pipes of the module with thermal insulation is visible.

## Figures and Tables

**Table 1:** Main parameters of the SIS100 accelerator superconducting busbar.

Parameter	Unit	Value
Cable diameter	mm	8.29
Tube inner diameter	mm	4.7
Strand pitch	mm	47
Sc. strand diameter	mm	0.8
Number of strands		23
Critical cable current	kA	21
Nominal cable current	kA	13.1
Current ramp rate	kA/s	26.2

**Table 2:** The chemical composition of the superconducting NbTi [%].

Cu	Mg	Al	Nb	Ni	Ti
98.15	-	1.15	0.36	0.09	0.07

**Table 3:** The mechanical properties of the superconducting wire from tensile test at room temperature. E—Young modulus, Rp0.1, Rp0.2 and Rp0.5—the tensile stress at 0.1, 0.2 and 0.5% of plastic strain respectively, Rm—ultimate stress.

Sample	E	Rp0.1	Rp0.2	Rp0.5	Rp0.2/Rm
	[GPa]	[MPa]	[MPa]	[MPa]	[%]
SC1	74.7	380.0	473.1	676.5	53.9
SC2	81.1	365.7	454.8	656.1	63.1
SC3	78.4	370.7	462.6	666.4	52.1
average	78.1	372.1	463.5	666.3	56.4

**Table 4:** The chemical composition of the cooling tube [%].

Cu	Ni	Si	Mn	Fe	Zn
67.58	30.08	0.63	0.79	0.74	0.14

**Table 5:** The mechanical properties of the cooling tube from tensile test at room temperature.

E	Rp0.1	Rp0.2	Rp0.5	Rt0.5	Rp0.2/Rm
[GPa]	[MPa]	[MPa]	[MPa]	[MPa]	[%]
134	234	248	269	257	72

E—Young modulus, Rp0.1, Rp0.2 and Rp0.5—the tensile stress for 0.1, 0.2 and 0.5% of plastic strain respectively, Rm—ultimate stress.

## Data Availability

The data presented in the article are available from the corresponding author.

## Supplementary Materials

The following are available online at https://www.mdpi.com/1996-1944/14/5/1097/s1, Video S1: bending simulation animation.
